# Online adaptive MR‐guided SBRT versus CT‐based planning in pancreatic cancer: A single‐center dosimetric comparative study

**DOI:** 10.1002/acm2.70391

**Published:** 2025-12-11

**Authors:** Randa Kamel, Thierry Gevaert, Dirk Van den Berge, Mark De Ridder

**Affiliations:** ^1^ Faculteit Geneeskunde en Farmacie Vrije Universiteit Brussel, Jette Brussels Belgium; ^2^ Radiation Oncology Department Universitair Ziekenhuis Brussel Vrije Universiteit Brussel, Jette Brussels Belgium

**Keywords:** dosimetric comparative study, online adaptive radiotherapy, pancreatic cancer, stereotactic body radiotherapy

## Abstract

**Background:**

Stereotactic body radiotherapy (SBRT) for pancreatic cancer is limited by the proximity of tumors to gastrointestinal (GI) organs, increasing the risk of toxicity. Magnetic resonance‐guided radiotherapy (MRgRT) offers potential advantages through superior soft tissue visualization, real‐time tumor tracking, and online adaptive planning. This study aimed to quantitatively compare dosimetric outcomes of pancreatic cancer SBRT using online adaptive MRgRT versus conventional computed tomography‐based image‐guided radiotherapy (CT‐IGRT).

**Methods:**

A retrospective dosimetric analysis was conducted on 100 plans from 10 patients with primary (*n* = 3) or recurrent (*n* = 7) pancreatic adenocarcinoma treated between July 2021 and December 2022 at UZ Brussel. Treatment included 80 adaptive MRgRT fractions, 10 initial MR plans, and 10 CT‐based volumetric‐modulated arc therapy (VMAT) plans using an internal target volume (ITV) approach. All patients were treated on the MRIdian system with daily online adaptation and real‐time beam gating. Dosimetric endpoints included target coverage, plan quality metrics, and organ‐at‐risk (OAR) doses. Statistical comparisons were performed using Wilcoxon signed‐rank tests.

**Results:**

MRgRT significantly reduced target volumes compared to CT‐IGRT (median gross tumor volume (GTV): 40.65 cc vs. 62.56 cc; *p* = 0.005; median planning target volume (PTV): 64.4 cc vs. 94.4 cc; *p* = 0.005) and improved dose coverage of both GTV and PTV. Intermediate dose spillage (R50%) was also lower with MRgRT (5.18 vs. 6.56, *p* = 0.04). MRgRT plans provided superior sparing of critical GI OARs, with median D_5cc_ relative dose reductions of 42% to the small bowel (*p* = 0.02), 23% to the duodenum (*p* = 0.02), and 13% to the stomach (*p* = 0.01). No significant differences were observed for the liver, kidneys, or large bowel. Treatment was well tolerated, with only grade I–II toxicities reported.

**Conclusions:**

MR‐guided adaptive SBRT demonstrated dosimetric superiority over CT‐IGRT in pancreatic cancer, with improved target coverage and enhanced GI OAR sparing. These findings support the use of MRgRT to expand the therapeutic window for safe dose escalation. Prospective studies are warranted to confirm clinical benefits.

## 1 INTRODUCTION

Pancreatic cancer is one of the most aggressive malignancies, with poor prognosis and limited curative options. While surgical resection offers the only potential cure, the majority of patients present with unresectable disease at diagnosis.^1^ Historically, conventionally fractionated radiotherapy has provided suboptimal outcomes, with median progression‐free survival (PFS) of approximately 16 months in the postoperative setting and around 10 months for unresected primaries.^2^, ^3^ Local progression is a significant cause of morbidity due to its association with high symptom burden, highlighting the need for an effective, durable, and safe local treatment modality.

Emerging evidence suggests that stereotactic body radiotherapy (SBRT), delivering high biologically effective doses (BED_10_, *α*/*β* = 10) > 70 Gy, could overcome the resistant nature of this disease and offer improved local control and survival outcomes.^4^, ^5^ However, unlike its well‐established role in lung and liver tumors, the adoption of SBRT in pancreatic cancer in any treatment setting remains limited. This is primarily due to the tumor's proximity to the luminal structures with concerns over accurate dose delivery and the risk of severe gastrointestinal (GI) toxicities.

A retrospective study conducted by Jolissaint et al. (2021) at Memorial Sloan Kettering Cancer Centre compared outcomes of surgical resection versus SBRT following induction chemotherapy for T4 pancreatic cancer. Both modalities showed comparable locoregional control and major GI toxicity rates at 25%, with SBRT delivered at BED_10_ doses > 97 Gy using hyper‐fractionated regimens (3–4.5 Gy per fraction).^6^

Conventional computed tomography‐based image‐guided radiotherapy (CT‐IGRT) for pancreatic SBRT is limited by poor soft tissue contrast, making it difficult to accurately visualize the pancreas and adjacent critical organs‐at‐risk (OARs) such as the duodenum, stomach, and bowel. As a result, larger planning target volume (PTV) margins, typically 5 mm or more, are often required to account for setup uncertainty, organ motion, and inter‐fractional anatomical variation, thereby increasing the risk of radiation‐induced toxicity.

In contrast, magnetic resonance‐guided radiotherapy (MRgRT) addresses these challenges by providing superior soft tissue visualization, real‐time tumor tracking, and daily online adaptive planning. These capabilities allow for reduced PTV margins and improved sparing of adjacent OARs, while maintaining or even enhancing target dose coverage. MRgRT has demonstrated feasibility and favorable dosimetric outcomes in abdominal malignancies, including pancreatic cancer, and is increasingly being adopted in this challenging clinical setting.^7^, ^8^, ^9^, ^10^

At our institution, MRgRT is delivered using the MRIdian linear accelerator (ViewRay Systems, Ohio, USA), which integrates continuous soft tissue tracking, automated beam gating, and online adaptive planning. These capabilities allow for reduced target volumes and improved dose sparing to the organs at risk (OARs). To quantify the dosimetric benefits of this technique, we conducted a comparative analysis between SBRT using CT‐IGRT versus adaptive MRgRT in the treatment of primary and recurrent pancreatic cancers.

This study analyzes dosimetric data from 80 treatment fractions and a total of 100 treatment plans across 10 patients treated with MRgRT at UZ Brussel between July 2021 and December 2022.

## 2 MATERIALS AND METHODS

Ten patients with biopsy‐proven pancreatic adenocarcinoma were included in this retrospective, single‐institution study; seven patients presented with local recurrences post‐surgical resection and adjuvant chemotherapy, while the remaining three were treated for inoperable locally advanced primary tumors following neoadjuvant chemotherapy. Our institution's ethical committee approval was granted on April 12, 2023, EC‐2023‐107. A detailed description of the patient cohort is provided in Table 1.

**TABLE 1:** Patient's characteristics.

Cohort no.	10 Patients
Median age at RT start	70 (54–81)
Sex	
Males	4 (40%)
Females	6 (60%)
Eastern Cooperative Oncology Group (ECOG) Performance Status scale	
1	9 (90%)
2	1 (10%)
Histology	
Adenocarcinoma	10 (100%)
Tumor recurrences	7 (70%)
Primaries	3 (30%)
Metastatic at diagnosis	
Yes	2 (20%)
No	8 (80%)
Tumor size	5.01 cm (3.00–6.48 cm)
Tumor volume	40.57 cc (13.88–67.12 cc)
Prior treatments to RT	
Surgery	7 (70%)
Chemotherapy	9 (90%)
Median prescribed dose	50 Gy (30–50 Gy)
Median number of fractions	5 Fractions (5–15 fractions)
Median BED_10_	100 Gy (45–100 Gy)

Abbreviations: BED_10_ biologically effective dose; RT, radiotherapy.

### 2.1 Study design

We compared here dosimetry data from two arms:

1. The first arm consisted of the actual delivered treatment plans using online adaptive MRgRT with automated beam gating, where the beam was delivered during the exhale phase.
2. The second arm included virtual parallel volumetric‐modulated arc therapy (VMAT) plans simulating CT‐IGRT. These were generated using our institution's standard CT‐guided pancreatic cancer SBRT protocol, based on 4D‐CT‐derived internal target volumes (ITVs).

Most prescriptions included two‐dose levels, with a median dose of 50 Gy to the gross tumor volume (GTV) (range: 30–50 Gy) and a median BED_10_ of 100 Gy (range: 45–100 Gy) delivered in five fractions (range: 5–15 fractions). The median tumor size was 5.01 cm at greatest dimension (range: 3.00–6.48 cm) and the median GTV volume, measured on simulation MRI, was 40.57 cc (range: 13.88–67.12 cc).

### 2.2 Treatment simulation

Patients were instructed to fast for 2 h prior to simulation and before every treatment session to minimize stomach volume and increase its separation away from the target. All patients were simulated in supine position with arms above the head using the Orfit AIO solutions cushions without abdominal compression.

A True Fast Imaging with Steady State Precession (TrueFISP) sequence was utilized for a 0.35T MRI simulation scan, enabling breath‐hold acquisitions as short as 17 s. Additionally, cine 2D acquisitions captured sagittal slices at 4–8 Hz during irradiation. Multiple breath‐hold scans were acquired in both inhalation and exhalation phases to determine the most convenient breath‐hold phase for each patient.

Each patient underwent four simulation CT simulation scans in an identical setup to the simulation MRI:

1. A free‐breathing, non‐contrast CT scan to allow conventional planning and treatment, if needed.
2. Two non‐contrast CT scans acquired in the same breath‐hold phase during simulation MRI for electron density information.
3. A contrast‐enhanced 4D‐CT for evaluation of the respiratory motion pattern.

All scans were acquired using a dual‐energy CT scanner (Revolution, GE Healthcare) with 3 mm slice thickness and 0.98 mm in‐plane image resolution. All scans;MRIs and CTs and the reconstructed 4D‐CT studies, were imported into MIM software (MIM Software Inc., Cleveland, OH) for contouring. The peak‐to‐peak motion amplitude on 4D‐CT was defined as the maximum displacement of the GTV between inhalation and exhalation phases.

### 2.3 Target volumes and organs at risk contouring

A radiation oncologist specializing in abdominal tumors contoured the target volumes and the OARs on the simulation MRI. Due to real‐time tumor tracking and automated beam gating, a minimal PTV of 3.0 mm was used as a direct isotropic expansion from the GTV. This margin was adopted from the ViewRay's stereotactic magnetic resonance‐guided adaptive radiation therapy (SMART) trial protocol for borderline resectable and locally advanced pancreatic cancer.^11^

OARs of comparative interest were the duodenum, stomach, small and large intestines, liver, and kidneys. All contours were peer‐reviewed during daily staff meetings prior to treatment delivery. Online adaptive contouring during treatment was performed in most fractions by the same physician to ensure consistency.

For data collection in this study, MR simulation scans, along with all daily fractional setup MRIs, were imported into the MIM software (MIM Software Inc., Cleveland, OH). All OARs were reviewed and, if necessary, edited by a treatment‐site specialized physician. A standard contouring margin of 3.0 cm isotropic expansion from the PTV was applied for reviewing OARs, except for the liver, kidneys, and stomach, which were contoured as whole organs regardless of proximity to the PTV.

The simulation 4D‐CT scan was used to replicate the structure sets for generating CT‐IGRT VMAT plans. A stable end‐of‐exhale phase was selected as the reference for OAR contouring. The GTV was delineated on each 4D‐CT phase using soft tissue window settings, and an internal target volume (ITV) was generated. A 3.0 mm isotropic expansion from the ITV was applied to create the PTV. Although our institution typically uses a 5.0 mm ITV‐to‐PTV expansion for CT‐IGRT‐based pancreatic SBRT, a 3.0 mm margin was used in this study to match the MRgRT plans and allow for direct dosimetric comparison.

### 2.4 Contouring and planning

All target and OAR contours were exported to the MRIdian treatment planning system (ViewRay Systems, Ohio, USA). The simulation breath‐hold CT scan was also transferred and deformably registered to the simulation MR scan to provide electron density data for accurate dose calculation.

The MRIdian linear accelerator delivers treatment using step‐and‐shoot intensity‐modulated radiation therapy (IMRT). Plans were generated using 18–21 beams, with 2.0 mm resolution Monte Carlo dose calculation incorporating magnetic field corrections.

### 2.5 On‐table adaptive workflow

An on‐table adaptive MRgRT workflow was followed for each treatment fraction. GTV and OAR contours from the simulation MRI were registered to the daily setup MRI. Within a region of interest extending 3 cm radially and 2 cm cranio‐caudally from the PTV, all contours were manually adjusted to reflect the day's anatomy as needed.

After recontouring, the original treatment plan was recalculated using the updated anatomy to generate a predicted dose distribution. If predicted doses violated dose constraints or compromised target coverage, the plan was reoptimized online to meet dosimetric goals before delivery.

### 2.6 CT‐based planning and non‐adaptive dose assessment

For CT‐based replanning, the Eclipse treatment planning system (Varian Medical Systems, Palo Alto, CA) was used. The same simulation breath‐hold CT was employed to ensure consistency in baseline dose calculations. Treatment plans were generated using four full VMAT arcs, with a calculation resolution of 2.5 mm, to meet dose constraints and achieve optimal coverage.

To assess the dosimetric impact of anatomical changes during treatment, the MRIdian TPS‐generated synthetic CT datasets from daily MR images acquired for MRgRT adaptation. These synthetic CTs enabled accurate dose recalculation based on the patient's anatomy for each treatment fraction.

The initial (non‐adaptive) CT‐based dose distribution was then mapped retrospectively onto each adaptive CT scan. This allowed for the estimation of the dose that would have been delivered had the original plan been used throughout treatment, without adaptation. By comparing these projected non‐adaptive doses to the actual adaptive doses delivered, we evaluated the clinical effectiveness of the adaptive MRgRT workflow in preserving target coverage and sparing the OARs.

### 2.7 Metrics of interest and organs at risk constraints

Dose constraints for abdominal SBRT were adopted from the UK consensus on normal tissue dose constraints for stereotactic radiotherapy and are summarized in Table 2.^12^ OAR metrics of interest included:

1. D_0.03cc_ and D_5cc_ for the duodenum, stomach, and small intestine.
2. D_0.03cc_ and D_20cc_ for the large intestine.
3. Mean dose to the liver and both kidneys.

**TABLE 2:** Dose constraints to the OARs and the frequency of their violations in MR‐guided adaptive radiation versus CT‐based VMAT plans.

OAR Dose_Volume_	Constraint (Gy)	MRgRT	CT‐IGRT
Duodenum D_0.03cc_	≤35	50%	67.5%
Duodenum D_5cc_	≤25	38.75%	67.5%
Small intestine D_0.03cc_	≤35	40%	58.75%
Small intestine D_5cc_	≤25	37.5%	58.75%
Stomach D_0.03cc_	≤35	0%	5%
Stomach D_5cc_	≤25	0%	2.5%
Large intestine D_0.03_	≤35	0%	1.25%
Large intestine D_20cc_	≤25	0%	1.25%
Liver D_mean_	<15	0%	0%
Bilateral kidneys D_mean_	<10	0%	0%

Abbreviations: CT, computed tomography; OAR, organ‐at‐risk; VMAT, volumetric‐modulated arc therapy.

The target coverage and plan quality metrics included:

1. Relative volumes receiving 95%, 90%, and 80% of the prescription dose (Rx).
2. Mean doses of the GTV/ITV and PTV.

Additional plan conformality and quality indices:

1. Homogeneity index (HI) of the PTV is calculated as dose to 2% volume to the PTV (D2%) to the dose to 98% (D98%).
2. Prescription isodose to target volume (PITV) ratio defined as the ratio of the Rx isodose volume (PI) to the target volume (TV), i.e., V100% Rx isodose/VPTV.
3. Gradient index (R50%), calculated as the volume of the 50% isodose line to the PTV volume.

### 2.8 Magnetic resonance‐guided treatment delivery and adaptation

Tumor tracking during treatment was performed using MR cine acquisition in the sagittal plane at 8 frames per second. The radiation beam was automatically paused if the tracked region of interest (ROI) moved outside a 3.0 mm gating boundary.

The GTV was aligned to the daily MR setup scan by initially rigidly copying the target contours from the MR simulation scan, then manually adjusted as needed. OARs were deformably registered and manually adjusted to account for anatomical differences. The PTV was then automatically re‐expanded based on the adapted contours.

The predicted dose (i.e., initial plan recalculated on the day's anatomy) was evaluated for target coverage and OAR constraint compliance. If the plan failed to meet clinical goals, re‐optimization was performed using the Monaco planning system to achieve the safest acceptable coverage. Upon approval by the attending physician and completion of physics QA, the adapted plan was delivered. All patients in this cohort underwent daily online adaptation, on average, treatment sessions lasted about 50–60 min.

### 2.9 Plans registration and comparison

Each daily adaptive MRgRT plan was compared in two ways: once against the initial MRgRT plan (predicted dose) and against the CT‐IGRT VMAT plans generated from the simulation CT scan, as previously described. All daily fractional setup MRIs, along with their final adapted structure sets, were imported into MIM software. These were registered to the CT‐IGRT plans, which were recalculated without re‐optimization. All dosimetric parameters of interest were then extracted and compared with the corresponding values from the initial plans.

### 2.10 Statistical analysis

Statistical analysis was performed using IBM SPSS Statistics (Version 29.0.2.0). Wilcoxon signed‐rank tests compared median dosimetric parameters and OAR doses on a per‐patient basis, with statistical significance defined as *p* ≤ 0.05.

## 3 RESULTS

Table 3 shows all median and range values of the parameters of interest, detailed above in the Methods section.

**TABLE 3:** Gated MR‐guided radiotherapy versus free‐breathing CT image‐guided radiotherapy.

Parameter	MRgRT median (range)	CT‐based FB IGRT median (range)	p‐Value
GTV/ITV volume in cc	40.57 cc (13.88–67.12 cc)	62.56 cc (29.00–107.67 cc)	*p* = 0.005
PTV volume in cc	64.41 cc (25.53–102.26 cc)	94.38 cc (47.99–151.28 cc)	*p* = 0.005
PITV (PTVD2%/D98%)	1.25 (1.00–2.60)	1.48 (1.00–2.60)	NS *p* = 0.20
HI (V100% Rx isodose/VPTV)	1.63 (1.20–1.90)	1.68 (1.29–6.39)	NS *p* = 0.20
R50% (50% Rx isodose/PTV)	5.18 (1.00–5.80)	6.56 (1.00–7.91)	*p* = 0.04
GTV/ITV_95%_ in Gy	38.31 Gy (34.56–49.18 Gy)	30.23 Gy (26.38–47.27 Gy)	*p* = 0.005
GTV/ITV_90%_ in Gy	42.88 Gy (36.17–49.74 Gy)	32.05 Gy (30.07–49.55 Gy)	*p* = 0.005
GTV/ITV_80%_ in Gy	48.24 Gy (37.86–50.11 Gy)	39.64 Gy (31.05–51.02 Gy)	*p* = 0.04
GTV/ITV_mean_ in Gy	49.17 Gy (39.59–50.75 Gy)	44.84 Gy (37.34–50.94 Gy)	*p* = 0.02
PTV_95%_ in Gy	32.54 Gy (27–44.71 Gy)	29.91 Gy (16.84–42.47 Gy)	NS *p* = 0.09
PTV_90%_ in Gy	35.68 Gy (29.11–46.09 Gy)	30.26 Gy (28.19–44.97 Gy)	NS *p* = 0.06
PTV_80%_ in Gy	42.71 Gy (32.42–48.16 Gy)	34.06 Gy (30.70–48.43 Gy)	*p* = 0.04
PTV_mean_ in Gy	47.36 Gy (36.99–49.51 Gy)	42.54 Gy (35.88–49.89 Gy)	*p* = 0.04
Duodenum D_0.03cc_ in Gy	36.79 Gy (22.77–48.26 Gy)	37.00 Gy (28.33–50.16 Gy)	NS *p* = 0.60
Duodenum D_5cc_ in Gy	23.23 Gy (16.80–39.00 Gy)	30.15 Gy (19.38–39.00 Gy)	*p* = 0.02
Small intestine D_0.03cc_ in Gy	29.97 Gy (10.03–41.77 Gy)	33.06 Gy (11.40–50.47 Gy)	*p* = 0.02
Small intestine D_5cc_ in Gy	17.72 Gy (4.49–28.89 Gy)	29.64 Gy (5.80–36.36)	*p* = 0.02
Stomach D_0.03cc_ in Gy	22.11 Gy (1.05–29.63 Gy)	24.48 Gy (1.20–30.50 Gy)	NS *p* = 0.30
Stomach D_5cc_ in Gy	12.53 Gy (0.83–18.10 Gy)	14.34 Gy (0.91–19.43 Gy)	*p* = 0.01
Large intestine D_0.03_ in Gy	21.27 Gy (11.31–36.66 Gy)	23.40 Gy (11.00–33.69 Gy)	NS *p* = 0.70
Large intestine D_20cc_ in Gy	11.63 Gy (7.28–24.00 Gy)	12.65 Gy (7.50–16.80 Gy)	NS *p* = 0.80
Liver D_mean_ in Gy	1.53 Gy (0.66–4.74 Gy)	1.59 Gy (0.67–5.88 Gy)	NS *p* = 0.30
Bilateral kidneys D_mean_ in Gy	3.40 Gy (2.22–5.90 Gy)	4.39 (3.05–7.10)	NS *p* = 0.10

Abbreviations: CT, computed tomography; IGRT, image‐guided radiotherapy; MRgRT, magnetic resonance‐guided radiotherapy; NS, non‐significant.

We analyzed dosimetry data from 100 plans, as follows: each of our 10 patients had an initial MR plan (10 plans) and a parallel CT‐IGRT plan (10 plans) added to 80 daily delivered adaptive MRgRT plans, where 7 patients received 5‐fraction courses (35 plans) and 3 patients received 15‐fraction courses (45 plans).

### 3.1 Target volume reduction and conformality

Analysis showed that the MR plans significantly reduced target volumes; the median GTV was 40.57 cc versus CT 62.56 cc (*p* = 0.005) and the median PTV was 64.41 cc versus CT 94.38 cc (*p* = 0.005). The HI and PITV were non‐significantly (NS) different. The R50% (intermediate dose spillage) favored MR plans: 5.18 versus 6.56 (*p* = 0.04). GTV/ITV coverage was significantly higher in MR versus CT plans across all thresholds (95%, 90%, 80%, and mean) (*p* = 0.005–0.04). PTV 80% and mean PTV coverage also improved (*p* = 0.04); PTV 95% and mean PTV approached significance (*p* = 0.09 and 0.06).

### 3.2 OARs sparing

Online adaptive MRgRT did significantly better in sparing the critical OARs; duodenum D_5cc_ received a median dose of 23.23 Gy versus 30.15 Gy (*p* = 0.02). D_0.03cc_ and D_5cc_ of the small intestine were both significantly lower at 29.97 Gy versus 33.06 Gy (*p* = 0.02) and 17.72 Gy versus 29.64 Gy (*p* = 0.02), respectively. Though dose constraint violations were not as significant for the stomach as they were for the duodenum and small intestine in the CT‐IGRT plans as shown in Table 2, still, dose reduction was significant for D_5cc_ at 12.53 Gy versus 14.34 Gy (*p* = 0.01). Relative dose reduction to the small intestine was 42%, duodenum 23%, and stomach 13%. No significant differences were found in doses delivered to the kidneys, liver, and large intestine.

**FIGURE 1:**
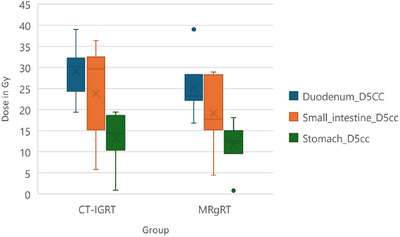
Boxplot representing dose differences delivered to the 5 cc volume of each of the duodenum, stomach, and small intestine through MRgRT versus CT‐IGRT. The *Y*‐axis represents the dose value in Gy. CT‐IGRT, computed tomography‐based image‐guided radiotherapy; MRgRT, magnetic resonance‐guided radiotherapy.

**FIGURE 2:**
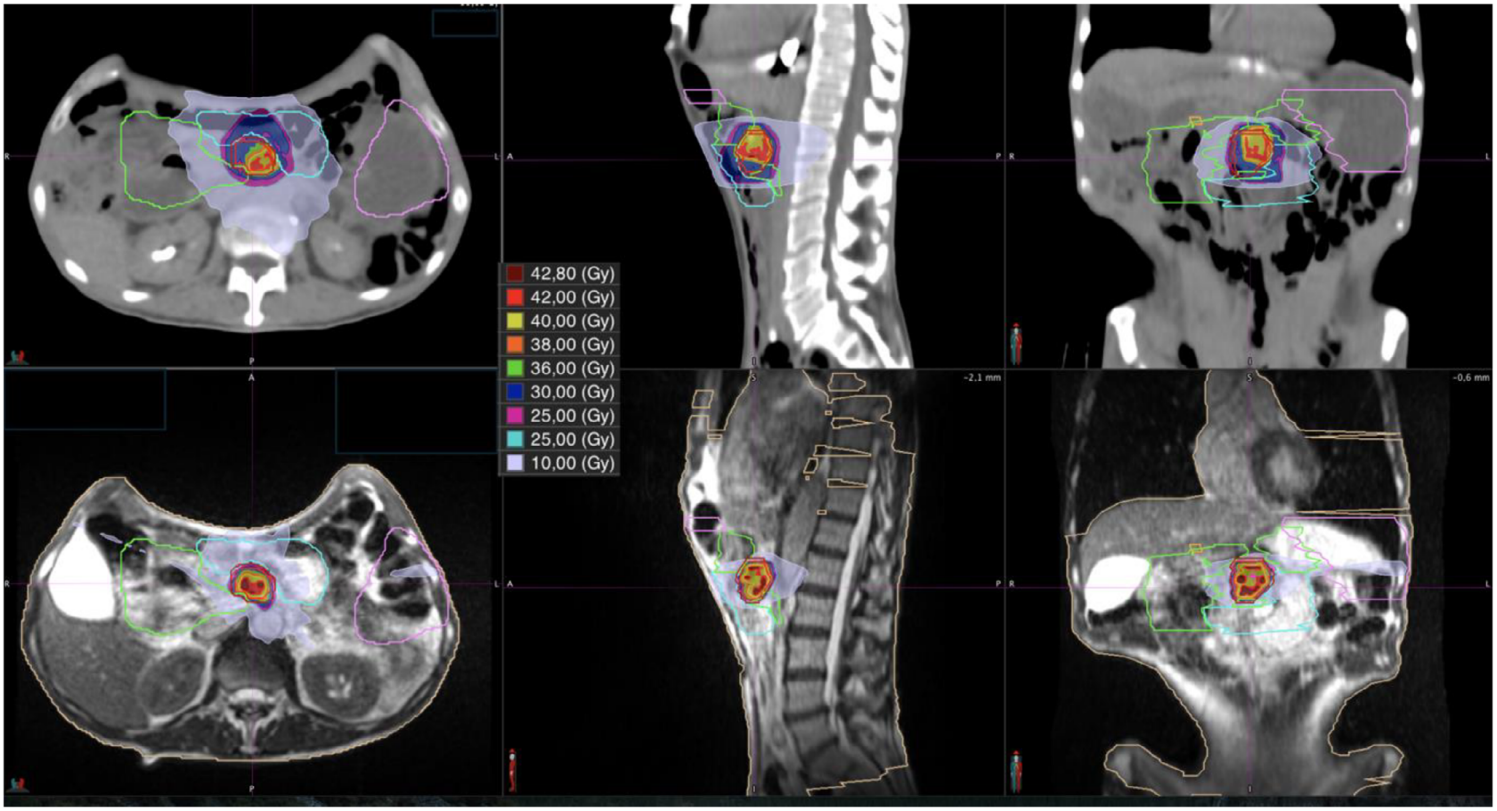
Representative treatment planning screenshots in axial, sagittal, and coronal planes showing dose distribution overlays for the same patient treated with MRgRT (bottom row) versus a virtual CT‐IGRT plan (top row). This figure serves as a visual illustration of the difference in soft tissue contrast between the two modalities and its potential impact on target delineation and OAR sparing. CT‐IGRT, computed tomography‐based image‐guided radiotherapy; MRgRT, magnetic resonance‐guided radiotherapy; OAR, organ‐at‐risk.

Figure 1 presents a quantitative comparison of D_5cc_ values for the duodenum, small intestine, and stomach between MRgRT and CT‐IGRT plans. To complement this, Figure 2 shows a representative patient case with side‐by‐side dose distribution overlays from MRgRT and CT‐IGRT plans. This qualitative illustration emphasizes the improved soft tissue contrast and dose shaping achievable with MR guidance, which may contribute to better target delineation and organ‐at‐risk sparing.

### 3.3 Treatment‐related toxicity

The median follow‐up was 24 months from the last patient receiving the last radiotherapy session. In terms of treatment‐related toxicity, only Grade I–II nausea and fatigue (CTCAE v5.0) were reported either acutely or as a late side effect. One patient developed acute biliary and duodenal obstruction during treatment, this was after receiving only two treatment sessions and this complication was attributed to clear tumor progression on imaging rather than a treatment side effect. That patient resumed RT after stenting and treatment was completed without any other reported adverse events.

## 4 DISCUSSION

The role of SBRT in pancreatic cancer continues to evolve, particularly in the lack of level I evidence and the ongoing variability in dose prescription practices. These challenges stem largely from the technical complexities associated with precise high‐dose delivery near the adjacent, radiosensitive GI structures in this patient population already burdened with a poor prognosis.^13^ Emerging evidence suggests that escalating the BED can improve local control and OS in pancreatic cancer, provided meticulous attention is paid to sparing the adjacent OARs.^14^, ^15^ For instance, a pooled analysis of 39 studies concluded that local control improved when BED_10_ exceeded 54 Gy, but the incidence of grade ≥ 3 GI toxicity increased substantially above 79.2 Gy.^16^ More recent investigations have demonstrated the feasibility of delivering even higher doses; Reyngold et al. used up to 98 Gy, and more recently, Jolissaint et al. reported comparable SBRT outcomes to those achieved with surgical resection, the current standard for curative intent, in selected patients, with similar toxicity profiles.^6^, ^17^

MRgRT has emerged as a promising modality in this context. Its superior soft tissue visualization, combined with real‐time tumor tracking and online adaptive planning makes it advantageous in delivering SBRT to abdominopelvic malignancies, particularly pancreatic cancer.^18^, ^19^, ^20^, ^21^ However, MRgRT is a resource‐intensive technique. Treatment times are longer due to adaptive re‐planning and gating workflows, requiring specialized personnel and dedicated infrastructure. This raises the need to evaluate whether such resource demands are justified by measurable dosimetric or clinical advantages across different disease sites. Rodriguez et al. quantitatively compared CT‐ versus MR‐guided RT for treating adrenal malignancies and concluded that MRgRT offered unmatched sparing of OARs.^22^

In our study, MRgRT enabled tighter margins, improved target coverage, and more effective sparing of the duodenum, stomach, and small intestine compared to simulated CT‐IGRT plans. Doses to these adjacent OARs were significantly reduced, with 5 cc relative dose reductions of up to 42% for the small intestine, while maintaining comparable conformality. The rate of OAR dose constraint violations observed with CT‐IGRT was up to 67.5%.

Importantly, both MRgRT and CT‐IGRT were delivered under free‐breathing conditions in this cohort. However, their approaches to motion management differed significantly. MRgRT utilized real‐time tumor tracking at frame rates of up to eight frames per second, enabling continuous intra‐fraction motion monitoring and automated beam gating delivered specifically during the end‐exhale phase. In contrast, the CT‐IGRT approach relied solely on a pre‐treatment 4D‐CT simulation to define an ITV. This method captures only a snapshot of the respiratory cycle and may not accurately represent variable tumor motion throughout the entire treatment course, as previously reported.^22^

This study has several limitations. First, it is a retrospective, single‐institution analysis, which introduces potential bias and limits generalizability. Second, the cohort was clinically heterogeneous, including both recurrent and metastatic cases, which may not reflect outcomes in patients treated with definitive intent. Third, this cohort represents our institution's early experience with online adaptive MRgRT. Greater familiarity and workflow optimization may further improve outcomes. Finally, while exploratory time‐to‐event data were included for reference, the study was designed as a dosimetric comparison and not powered to assess clinical outcomes. Therefore, no statistical conclusions regarding efficacy should be drawn from these findings.

Prospective trials are needed to confirm whether the dosimetric benefits of MRgRT translate into improved clinical outcomes, such as local control, toxicity reduction, and survival. Such studies will also be essential for refining patient selection, evaluating cost‐effectiveness, and optimizing workflow strategies to guide the broader implementation of MRgRT in routine practice.

## 5 CONCLUSIONS

This dosimetric analysis highlights the potential of MR‐guided radiotherapy to improve the therapeutic index in pancreatic SBRT by enabling tighter margins, enhanced target coverage, and reduced dose to adjacent gastrointestinal organs. These gains, however, come with significant workflow and resource demands. As MRgRT becomes more widely available, understanding its true clinical value beyond dosimetric advantages will be essential. Prospective trials are needed not only to validate improvements in local control and toxicity, but also to clarify cost‐effectiveness and patient selection criteria that justify its broader adoption in routine practice.

## AUTHOR CONTRIBUTIONS

**Randa Kamel**: Conceptualization; investigation; methodology; data curation; formal analysis; writing—original draft. **Thierry Gevaert**: Investigation; methodology; project administration; resources; supervision; validation; visualization; writing—editing; reviewing. **Dirk Van den Berge**: Methodology; supervision; writing—editing; reviewing. **Mark De Ridder**: Supervision; writing—editing; reviewing.

## CONFLICT OF INTEREST STATEMENT

The authors declare no conflict of interest.

## ETHICS STATEMENT

Ethical committee (of the UZ Brussel) approval was granted on April 12, 2023 (EC‐2023‐107).
